# A longitudinal evaluation of fatigue in chronic inflammatory demyelinating polyneuropathy

**DOI:** 10.1002/brb3.2712

**Published:** 2022-07-21

**Authors:** Karissa L. Gable, Stojan Peric, Michael W. Lutz, Ivo Bozovic, Milutin Petrovic, Aleksandar Stojanov, Ivana Basta, Jeffrey A. Allen

**Affiliations:** ^1^ Duke Neurological Disorders Clinic Duke University Medical Center Durham North Carolina USA; ^2^ Faculty of Medicine and University Clinical Center of Serbia, Neurology Clinic University of Belgrade Belgrade Serbia; ^3^ Neurology Clinic University Clinical Center of Serbia Belgrade Serbia; ^4^ Neurology Clinic University Clinical Center of Kragujevac Kragujevac Serbia; ^5^ Neurology Clinic University Clinical Center of Nis Nis Serbia; ^6^ Department of Neurology University of Minnesota Minneapolis Minnesota USA

**Keywords:** chronic inflammatory demyelinating polyneuropathy, fatigue, quality of life

## Abstract

**Background and aims:**

Fatigue is a common but poorly understood complaint in patients with immune‐mediated polyneuropathies. We sought to evaluate changes in fatigue over 1 year in a cohort of chronic inflammatory demyelinating polyneuropathy (CIDP) patients and to correlate changes in fatigue with changes in disability and quality of life. Investigation into other factors that may contribute to fatigue with a particular interest in the role other chronic disease states may play was also performed.

**Methods:**

Fifty patients with CIDP who satisfied the 2010 EFNS/PNS diagnostic criteria were followed over the period of 1 year at three tertiary care centers in Serbia. Assessments of disability, quality of life, and patient perception of change and fatigue were collected at two time points 12 months apart. Comorbidities, treatment regimens, and sedating medication use was collected.

**Results:**

Disability, quality of life, and patient perception of change showed statistically significant correlations with change in fatigue (*p* < .01). Increased levels of fatigue were noted in patients who used sedating medications (*p* = .05) and who had a comorbid chronic medical condition (*p* = .01).

**Interpretation:**

Worsening fatigue correlates over time with increased disability and worse quality of life. Fatigue is not specific to CIDP, but is common in many chronic medical conditions and with the use of sedating medications. Our findings support the importance of identifying and supportively managing fatigue in patients with CIDP, but cautions against considering fatigue as a CIDP diagnostic symptom or using fatigue to justify immunotherapy utilization.

## Graphical abstract

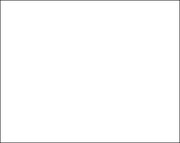

## 1 INTRODUCTION

Chronic inflammatory demyelinating polyneuropathy (CIDP) is an autoimmune peripheral nerve disease that is defined clinically by numbness and weakness that evolves over 2 months or more in a progressive or relapsing pattern (Joint Task Force of the European Federation of NeurologicalSocieties/Peripheral Nerve Society, 2010; Van den Bergh et al., 2021). Fatigue is a nonspecific symptom that is reported in up to 80% of patients with CIDP (Merkies et al., 1999). Frequently identified by patients as one of the most disabling symptoms that deleteriously impacts quality of life, (Bozovic et al., 2017) fatigue may be present in both active disease and when the disease is in pharmacologic remission (Gable et al., 2020). The frequent occurrence of fatigue in CIDP is not unique. It is common in many chronic medical illnesses and may be driven by both peripheral and central factors (Jiang et al., 2020; Paneroni et al., 2020). Peripherally, nerve, muscle, or neuromuscular junction pathology and the resulting weakness that ensues may be perceived as fatigue, while central fatigue is experienced as a lack of energy not related to muscle weakness. Many factors may contribute to fatigue. Peripheral fatigue may be influenced by the severity of functional disability, while central fatigue can be driven by sedating medications, poor sleep quality, and depression (Gable et al., 2020). Several unanswered questions remain with regard to CIDP and fatigue. Although the frequency of fatigue is well defined, how fatigue evolves over time in individual patients is less clear. The factors that influence fatigue in CIDP are also ill defined. We sought to better understand how fatigue changes over time in patients with CIDP, and what factors influence fatigue severity. By doing so we aim to better understand fatigue management strategies in patients with CIDP.

## 2 MATERIALS AND METHODS

### 2.1 Identification of patients

In this longitudinal prospective multicenter study, consecutive patients were enrolled at three tertiary care centers in Serbia between February 2017 and January 2019. All patients satisfied the 2010 European Federation of Neurological Societies/Peripheral Nerve Society (EFNS/PNS) CIDP diagnostic criteria for “definite,” “probable,” or “possible” CIDP (Joint Task Force of the European Federation of NeurologicalSocieties/Peripheral Nerve Society, 2010). Demographic data including age of CIDP onset, age at study enrollment, and gender were recorded. Comorbid medical conditions, immunotherapy medications, neuropathic symptomatic medications, and sedating medications were cataloged for each patient. We considered anticonvulsants (gabapentin, pregabalin, carbamazepine) and antidepressants (amitriptyline, duloxetine) as neuropathic medications. Opioids, benzodiazepines, and antispasmodic agents were classified as nonneuropathic sedating medications.

### 2.2 Standard protocol approval, registration, and patient consent

Written informed consent was obtained from every study participant. This study was conducted with the approval from the Ethical Committee of the Faculty of Medicine, University of Belgrade and with the institutional review board approval at each participating site.

### 2.3 Assessment of outcome measures

Outcomes were collected at baseline and at 12 months. Krupp's Fatigue Severity Scale (FSS) was used to evaluate fatigue severity (Krupp et al., 1989). The FSS is a nine‐item instrument designed to assess fatigue as a symptom of a variety of different chronic conditions. The scale is scored from 0 to 63 where higher scores indicate more fatigue, and a score above 36 means significant fatigue. The Inflammatory Neuropathy Cause and Treatment (INCAT) disability score (Hughes et al., 2008) and the Inflammatory Rasch‐built Overall Disability Scale (I‐RODS) (van Nes et al., 2011) were used to assess disability. This scale was adapted and validated in the Serbian population (Peric et al., 2019). Raw scores from the I‐RODS scale were converted to the centile score for analysis. The chronic acquired polyneuropathy patient‐reported index (CAP‐PRI) and patient perception in change (PGIC) were used to capture quality of life (Gwathmey et al., 2016). CAP‐PRI is a 15‐item questionnaire developed specifically for patients with chronic immune‐mediated polyneuropathies that assesses life quality across multiple domains. PGIC reflects a patient's belief about their health and efficacy of treatment. Participants were asked to “indicate how you feel now, compared to how you felt before receiving treatment” on a 7‐point scale ranging from +3 (very much improved) to −3 (very much worsened). Participants were also asked “how do you estimate your health” on a similar 7‐point scale. PGIC questions were only asked at the 12‐month assessment.

Patients were stratified into off‐immunotherapy or on‐immunotherapy as defined by CIDP disease activity status (CDAS) criteria: (Gorson et al., 2010) CDAS 1 “Cure” off immunotherapy for at least 5 years; CDAS 2 “Remission” off immunotherapy for less than 5 years; CDAS 3 “Stable active disease” on immunotherapy for at least 1 year; CDAS4 “Improvement” on immunotherapy for at least 3 months but less than 1 year; and CDAS 5 “Unstable active disease” immunotherapy naïve or initiated up to 3 months earlier. For the purposes of this analysis, CDAS classes 1 and 2 (off‐immunotherapy) were combined, as were CDAS classes 3, 4, 5 (on‐immunotherapy).

### 2.4 Statistical analysis

As the aim of this study was to evaluate changes in fatigue over a time period of 1 year and to correlate changes in fatigue with changes in disability and quality of life, the statistical analysis focused on changes in FSS, outcome measures, medication use and comorbidities between baseline and the 1‐year follow‐up visit. Descriptive statistics for FSS and outcome measure (retest‐baseline) were calculated. Repeated measures analysis of variance was used to evaluate the statistical significance of changes in FSS (retest compared with baseline for each subject) with respect to each of the outcome measures or conditions (comorbidities and medication use). A linear mixed model was fit to the data with FSS as the dependent variable and the outcome measures and conditions as predictors with subject as a random effect. Gender was included as a covariate and interactions between the outcome measures/conditions and time was assessed. Spearman's rho was calculated to assess the correlation between changes in continuous outcome measures and FSS.

## 3 RESULTS

### 3.1 Demographic and clinical data

Fifty patients were included in the analysis. Thirty‐one (62%) were classified as “typical” CIDP. Remaining patients were classified as sensory (14%), multifocal (12%), distal (8%), and motor (4%) variants. Forty‐four (88%) satisfied “definite” CIDP diagnostic criteria, while 2% were classified as probable and 10% as possible. The proportion of patients receiving immunotherapy (CDAS status 3, 4, 5) and those off immunotherapy (CDAS 1, 2) was similar at study onset (54% off‐immunotherapy) and at 12 month follow up (68% off‐immunotherapy). When considering only changes between CDAS 1, 2 and CDAS 3, 4, 5 categories, a total of five patients changed classification during the 12‐month study period: two patients moved from off‐immunotherapy to on‐immunotherapy and three patients were reclassified in the opposite direction. There was not a substantial mean change in FSS over 12 months (1.2), however there was considerably variability in change in FSS (SD = 16.2) with some individuals showing substantial improvement and others substantial decline.

### 3.2 Correlations with changes in fatigue

**FIGURE 1:**
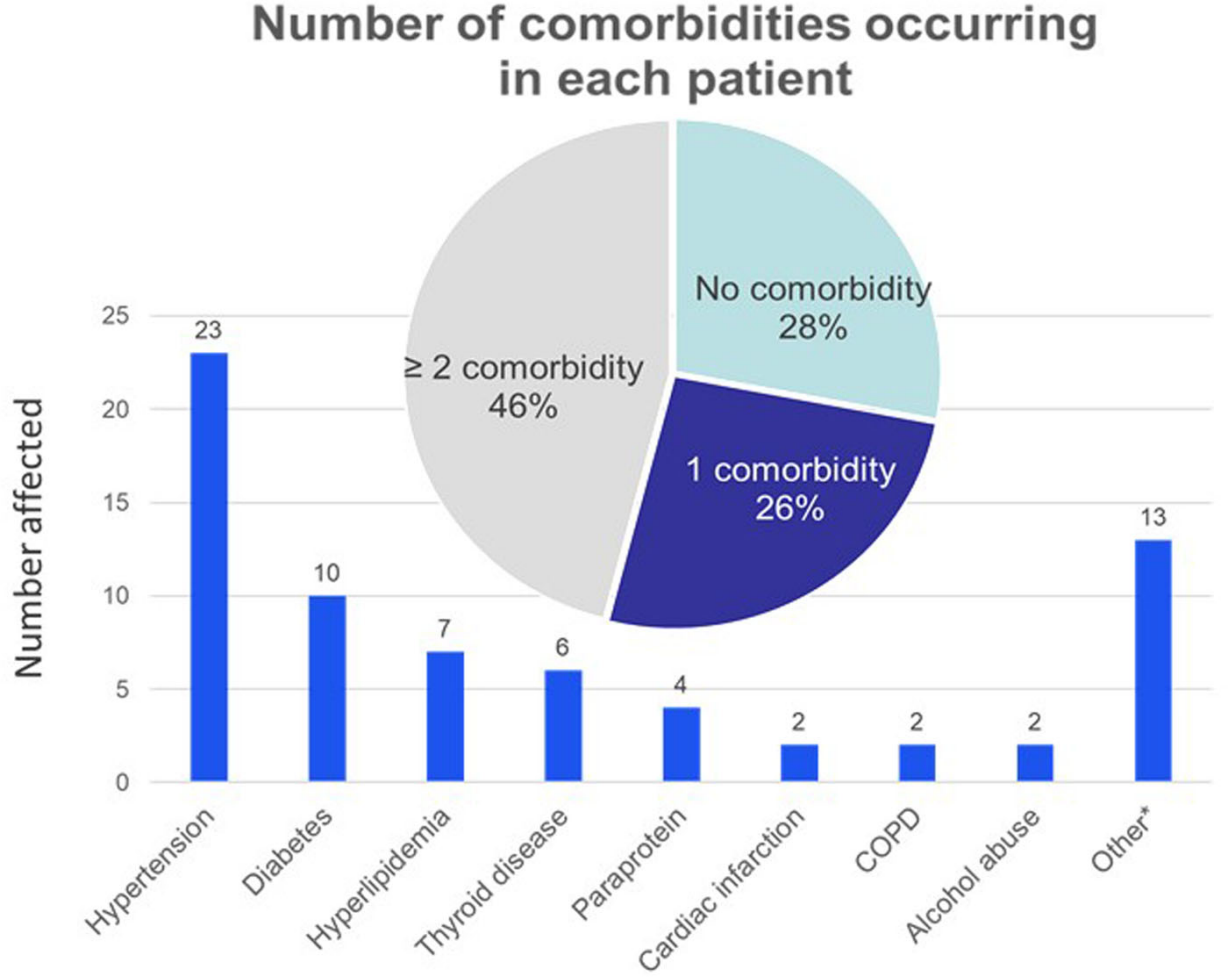
Number of comorbidities occurring in each patient with CIDP

Correlations between changes in fatigue and demographics, disability and quality of life are shown in Table 1. The correlation of FSS and I‐RODS at baseline was −0.62; the correlation of FSS and I‐RODS at 12 months was −0.65. Worsening fatigue correlated significantly with increased disability when assessed with the I‐RODS score (*p* < .01), but not INCAT disability scale. Fatigue also had a positive correlation with worse quality of life and worse patient perception of health over the course of 1 year (*p* < .01). A multitude of comorbid medical conditions were present, with nearly half (46%) having 2 or more medical illnesses unrelated to CIDP (Figure 1). CIDP treatments during the 1‐year study period included corticosteroids (46% of patients) and IVIG (18%). Sedating medications including medications to treat neuropathic pain (40%) as well as sedating medications not used for the treatment of pain (24%). were also frequently used. The presence of any comorbid illness correlated with fatigue worsening over the course of 1 year (*p* = .01) as did utilization of sedating medications (*p* = .05). Immunotherapy and neuropathic medications showed no correlation with fatigue (Table 2).

**TABLE 1:** Correlation between change in fatigue and demographics, disability, and quality of life

Outcome measure	Baseline, mean (SD)	Month 12, mean (SD)	Mean change within subject (SD)	*p*	Spearman, rho (*p*)
FSS	31.9 (16.4)	32.8 (14.2)	1.2 (16.2)	–	–
Presence of fatigue (FSS > = 36) (%)	40.8	40.0			
Age at enrollment, years (SD)	59.4 (14.0)	–		.77	.06 (.67)
Age disease onset, years (SD)	54.5 (14.9)	–		.41	−.14 (.32)
Female, *n* (%)	20 (40%)	–		.08	–
I‐RODS	65.8 (24.3)	61.1 (23.6)	−4.2 (7.8)	<.01	−.41 (<.01)
INCAT	1.67 (1.95)	2.1 (1.9)	0.6 (1.6)	.14	.10 (.49)
CAP‐PRI	9.7 (8.1)	13.4 (7.9)	1.1 (6.1)	<.01	−.66 (<.01)
CDAS 1, 2 (off immunotherapy), *n* (%)	26 (52%)	34 (68%)	8 (2%)	.92	–
PGIC perceived change in health over 1 year	2.9 (1.2)	3.1 (1.0)	0.2 (1.0)	.02	.43 (<.01)
PGIC perception of health	3.0 (1.0)	3.2 (1.1)	0.2 (1.1)	<.01	.52 (<.01)

**TABLE 2:** Correlation between fatigue and comorbidities

Condition	Mean difference in FSS if condition present, mean (SE)	Mean difference in FSS if condition absent, mean (SE)	*p*
Any comorbidity	2.7 (2.7)	−2.8 (4.5)	.01
Sedating medication	7.6 (3.0)	−0.84 (2.8)	.05
Neuropathic medication	1.0 (4.4)	−0.84 (2.8)	.94
Corticosteroids, pulsed	−3.1 (4.3)	2.1 (2.6)	.32
Corticosteroids, daily oral	−0.43 (4.1)	1.9 (2.8)	.65
IVIG	−9.3 (5.6)	3.3 (2.4)	.07
Any immune therapy	9.6 (3.8)	0.27 (2.5)	.08

## 4 DISCUSSION

In this prospectively collected study of patients with CIDP, we show that over the course of 1‐year higher levels of fatigue correlate with more disability and worse quality of life. We also demonstrate that fatigue in patients with CIDP is greater when other chronic medical illnesses are present and in the context of sedating medications. The findings reinforce the notion that fatigue is a nonspecific symptom that does not define CIDP, but rather can be a consequence of chronic diseases of many types (Jiang et al., 2020; Paneroni et al., 2020; Seifert & Baerwald, 2019; Swain & Jones, 2019). In CIDP, fatigue is present in patients with both active disease and in patients in remission (Gable et al., 2020). CIDP diagnostic criteria are careful to avoid inclusion of fatigue as a defining diagnostic characteristic (Joint Task Force of the European Federation of NeurologicalSocieties/Peripheral Nerve Society, 2010; Van den Bergh et al., 2021). In clinical practice, these findings collectively suggest fatigue is an important symptom that should be addressed with each patient, but that should not be used to guide diagnostic or immunotherapy paradigms. In summary, chronic immune therapy may improve patients’ functional impairment and disability, but will not be able to completely eliminate fatigue in the setting of chronic disease, whether active or in remission.

Patients in our study had worse quality of life as fatigue worsened over the course of a year, and also were more likely to perceive a decline in their overall health. The frequency of impaired quality of life in patients with CIDP has been well described. Patients with CIDP have consistently been shown to have lower baseline health‐related quality of life compared with historical controls (Allen et al., 2021; Gwathmey et al., 2016; Maxwell et al., 2013; Merkies et al., 2002; Padua et al., 2004). In one large survey of patients with unconfirmed CIDP, weakness and numbness in the lower limbs, poor balance, and fatigue were among the most bothersome symptoms reported by patients (Allen et al., 2021). While disease burden vis‐à‐vis weakness and sensory loss invariably contribute to poor quality of life, our findings highlight the important role that nonspecific fatigue plays as well. This association has important management implications. Although the best strategy to manage fatigue in CIDP is unknown, recognizing the source of fatigue may guide the most appropriate approach. Exercise has been shown to improve fatigue in most chronic disorders, although the effect specifically in CIDP is presently unproven (Jiang et al., 2020; Paneroni et al., 2020). Reviewing and minimizing sedating medications is an obvious but often overlooked intervention. Although not specifically studied in our analysis, optimization of sleep quality and depression management may be beneficial to some patients as well (Gable et al., 2020).

Our study has several limitations. Strength impairment outcomes, in particular with hand held grip strength dynamometer, were not consistently collected in this cohort. The absence of strength outcomes precludes our ability to longitudinally correlate changes in fatigue with strength impairment. Our patients were also geographically isolated to three centers in Serbia. Although we have no reason to believe that this population would be different from other groups throughout the world, local or cultural influencers of fatigue unique to this cohort cannot be excluded, and hence the applicability of our findings to other geographic areas is uncertain. It is notable that 52% % of patients in our cohort at the onset of the study period were classified as CDAS 1–2 (remission or cure, off all immunotherapy). This finding differs from the typical distribution of patients in drug free remission or cure, which has generally reported to be near 30% (Gorson et al., 2010). This observation likely reflects a generally conservative treatment approach in Serbia where immunotherapy is typically utilized for short periods of time (often months) during periods of clinical deterioration. Unlike other parts of Europe and in the USA, long‐term maintenance immunoglobin therapy for stable patients is rarely administered. Local access to immunoglobulin and intolerance to corticosteroids may contribute to these disparities as well. Additionally, our analysis did not include other important influencers of fatigue, including quantification of pain and depression. Although these factors have previously been shown to impact fatigue and quality of life, the extent to which they impact longitudinal change in our cohort is unknown.

Our findings add to the evolving understanding of fatigue in CIDP. While frequently a part of the disease, so too is it frequently part of many immune and nonimmune mediated chronic conditions. We caution placing an over‐emphasis on fatigue as a diagnostic symptom of CIDP, and instead encourage reliance on disease defining symptoms when making a clinical diagnosis of CIDP (Joint Task Force of the European Federation of NeurologicalSocieties/Peripheral Nerve Society, 2010; Van den Bergh et al., 2021). For the same reason, we also caution against using fatigue to justify immunotherapy management or when assessing response to treatment. Prior studies have highlighted the pitfalls of using nonspecific changes in subjective symptoms to justify long‐term immunotherapies (Allen & Lewis, 2015). Because of the frequency and often severity of fatigue in CIDP, we encourage clinicians to ask patents about fatigue with a particular focus on modifiable factors. Although further research is needed to understand the best management approach, opportunities for intervention may include optimization of activity and exercise, minimization of sedating medications, improvement of sleep quality, and treatment of depression. Improvement of fatigue through one or more of these pathways has the capacity to improve quality of life in CIDP patients independent of the immunologic disease state or use of immune‐based medications.

## CONFLICT OF INTEREST

K. G. was a consultant for Apellis and Medscape. J. A. is a consultant for Argenx, alexion, annexon, Akcea, CSL behring, Johnson & Johnson, Grifols, Sanofi, Takeda. S. P. reports the following: he has received lecture honoraria from Pfizer, Viatris, Teva Actavis, Berlin Chemie Menarini, Mylan, Worwag, Adoc and Salveo; research grants from Kedrion, Octapharma and Argenx; consultant fees from Argenx, Mylan and Roche; and travel grants from Octapharma, Kedrion, Teva Actavis, Sanofi Genzyme, Pfizer, Roche, Adoc and Berlin Chemie Menarini. The remaining authors have no disclosures to report.

### PEER REVIEW

The peer review history for this article is available at https://publons.com/publon/10.1002/brb3.2712

## Data Availability

The data support the findings of this study and are available from the corresponding author upon reasonable request.

## References

[brb32712-bib-0019] Allen, J. A. , Levine, T. , Butler, L. , & Haudrich, A. (2021). A global survey of disease burden in patients who carry a diagnosis of chronic inflammatory demyelinating polyneuropathy. Advances in Therapy, 38(1), 316–328. 33113101 10.1007/s12325-020-01540-6 PMC7854453

[brb32712-bib-0020] Allen, J. A. , & Lewis, R. L. (2015). CIDP diagnostic pitfalls and perception of treatment benefit. Neurology, 85(6), 498–504. 26180143 10.1212/WNL.0000000000001833

[brb32712-bib-0004] Bozovic, I. , Kacar, A. , Peric, S. , Nikolic, A. , Bjelica, B. , Cobeljic, M. , Petrovic, M. , Stojanov, A. , Djuric, V. , Stojanovic, M. , Djordjevic, G. , Martic, V. , Dominovic, A. , Vukojevic, Z. , & Basta, I. (2017). Quality of life predictors in patients with chronic inflammatory demyelinatingpolyradiculoneuropathy. Journal of Neurology, 264(12), 2481–2486. 29086018 10.1007/s00415-017-8658-x

[brb32712-bib-0005] Gable, K. L. , Attarian, H. , & Allen, J. A. (2020). Fatigue in chronic inflammatory demyelinating polyneuropathy. Muscle & Nerve, 62(6), 673–680. 32710648 10.1002/mus.27038

[brb32712-bib-0013] Gorson, K. C. , van Schaik, I. N. , Merkies, I. S. , Lewis, R. A. , Barohn, R. J. , Koski, C. L. , Cornblath, D. R. , Hughes, R. A. C. , Hahn, A. F. , Baumgarten, M. , Goldstein, J. , Katz, J. , Graves, M. , Parry, G. , & van Doorn, P. A. (2010). Chronic inflammatory demyelinating polyneuropathy disease activity status: Recommendations for clinical research standards and use in clinical practice. Journal of the Peripheral Nervous System, 15, 326–333. 21199104 10.1111/j.1529-8027.2010.00284.x

[brb32712-bib-0012] Gwathmey, K. G. , Conaway, M. R. , Sadjadi, R. , Joshi, A. , Barnett, C. , Bril, V. , Ng, E. , David, W. , Gable, K. , Guptill, J. T. , Hobson‐Webb, L. D. , Dineen, J. , Hehir, M. , Brannagan, T. H. 3rd , Byun, E. , Adler, M. , & Burns, T. M. (2016). Construction and validation of the chronic acquired polyneuropathy patient‐reported index (CAP‐PRI): A disease‐specific, health‐related quality‐of‐life instrument. Muscle & Nerve, 54(1), 9–17. 26600438 10.1002/mus.24985 PMC4950873

[brb32712-bib-0009] Hughes, R. A. , Donofrio, P. , Bril, V. , Dalakas, M. C. , Deng, C. , Hanna, K. , Hartung, H. ‐ P. , Latov, N. , Merkies, I. S. J. , & van Doorn, P. A. , ICE Study Group . (2008). Intravenous immune globulin(10% caprylate‐chromatography purified) for the treatment of chronic inflammatory demyelinating polyradiculoneuropathy (ICE study): A randomised placebo controlled trial. Lancet Neurology, 7, 136–144. 18178525 10.1016/S1474-4422(07)70329-0

[brb32712-bib-0006] Jiang, M. , Ma, Y. , Yun, B. , Wang, Q. , Huang, C. , & Han, L. (2020). Exercise for fatigue in breast cancer patients: An umbrella review of systematic reviews. International Journal of Nursing Sciences, 7(2), 248–254. 32685623 10.1016/j.ijnss.2020.03.001 PMC7355202

[brb32712-bib-0001] Joint Task Force of the European Federation of NeurologicalSocieties/Peripheral Nerve Society . (2010). European Federation of Neuro‐logical Societies/Peripheral Nerve Society Guideline on management of chronic inflammatory demyelinating polyradiculoneuropathy: Report of a joint task force of the European Federation of Neurological Societies and the Peripheral Nerve Society first revision. Journal of the Peripheral Nervous System, 15, 1–9. 10.1111/j.1529-8027.2010.00245.x 20433600

[brb32712-bib-0008] Krupp, L. B. , LaRocca, N. G. , Muir‐Nash, J. , & Steinberg, A. D. (1989). The fatigue severity scale. Application to patients with multiple sclerosis and systemic lupus erythematosus. Archives of Neurology, 46(10), 1121–1123. 2803071 10.1001/archneur.1989.00520460115022

[brb32712-bib-0018] Maxwell, S. K. , Barnett, C. , Kokoyi, S. , Leung, J. C. , Yu, J. J. , Bril, V. , & Katzberg, H. D. (2013). Association of social support with quality of life in patients with polyneuropathy. Journal of the Peripheral Nervous System, 18, 37–43. 23521642 10.1111/jns5.12005

[brb32712-bib-0003] Merkies, I. S. , Schmitz, P. I. , Samijn, J. P. , van der Meche, F. G. , & van Doorn, P. A. (1999). Fatigue in immune‐mediated polyneuropathies. European Inflammatory Neuropathy Cause and Treatment (INCAT) group. Neurology, 53, 1648–1654. 10563607 10.1212/wnl.53.8.1648

[brb32712-bib-0016] Merkies, I. S. , Schmitz, P. I. , van der Meché, F. G. , Samijn, J. P. , & van Doorn, P. , for the Inflammatory Neuropathy Cause and Treatment (INCAT) Group . (2002). Quality of life complements traditional outcome measures in immune‐mediated polyneuropathies. Neurology, 59, 84–91. 12105312 10.1212/wnl.59.1.84

[brb32712-bib-0007] Paneroni, M. , Vitacca, M. , Venturelli, M. , Simonelli, C. , Bertacchini, L. , Scalvini, S. , Schena, F. , & Ambrosino, N. (2020). The impact of exercise training on fatigue in patients with chronic obstructive pulmonary disease: A systematic review and meta‐analysis. Pulmonology, 26(5), 304–313. 32184070 10.1016/j.pulmoe.2020.02.004

[brb32712-bib-0017] Padua, L. , Aprile, I. , Caliandro, P. , Padua, R. , Mazza, S. , & Tonali, P. (2004). Intravenous immunoglobulin treatment in autoimmune neurological disorders: Pilot study on early effects on patients' quality of life. Journal of the Peripheral Nervous System, 9, 3–6. 14871448 10.1111/j.1085-9489.2004.09102.x

[brb32712-bib-0011] Peric, S. , Bozovic, I. , Pruppers, M. H. J. , Bjelica, B. , Stevic, Z. , Faber, C. G. , Merkies, I. S. J. , & Basta, I. (2019). Validation of the Serbian version of inflammatory Rasch‐built overall disability scale in patients with chronic inflammatory demyelinating polyradiculoneuropathy. Journal of the Peripheral Nervous System, 24(3), 260–267. 10.1111/jns.12343 31397933

[brb32712-bib-0014] Seifert, O. , & Baerwald, C. (2019). Impact of fatigue on rheumatic diseases. Best Practice & Research. Clinical Rheumatology, 33(3), 101435. 31703791 10.1016/j.berh.2019.101435

[brb32712-bib-0015] Swain, M. G. , & Jones, D. E. J. (2019). Fatigue in chronic liver disease: New insights and therapeutic approaches. Liver International, 39(1), 6–19. 29935104 10.1111/liv.13919

[brb32712-bib-0002] Van den Bergh, P. Y. K. , van Doorn, P. A. , Hadden, R. D. M. , Avau, B. , Vankrunkelsven, P. , Allen, J. A. , Attarian, S. , Blomkwist‐Markens, P. H. , Cornblath, D. R. , Eftimov, F. , Goedee, H. S. , Harbo, T. , Kuwabara, S. , Lewis, R. A. , Lunn, M. P. , Nobile‐Orazio, E. , Querol, L. , Rajabally, Y. A. , Sommer, C. , & Topaloglu, H. A. (2021). European Academy of Neurology/Peripheral Nerve Society guideline on diagnosis and treatment of chronic inflammatory demyelinating polyradiculoneuropathy: Report of a joint Task Force‐Second revision. Journal of the Peripheral Nervous System, 26(3), 242–268. 34085743 10.1111/jns.12455

[brb32712-bib-0010] van Nes, S. I. , Vanhoutte, E. K. , van Doorn, P. A. , Hermans, M. , Bakkers, M. , Kuitwaard, K. , Faber, C. G. , & Merkies, I. S. J. (2011). Rasch‐built Overall Disability Scale (R‐ODS) for immune‐mediated peripheral neuropathies. Neurology, 76, 337–345. 21263135 10.1212/WNL.0b013e318208824b

